# A New Mechanism of Vitamin C Effects on A/FM/1/47(H1N1) Virus-Induced Pneumonia in Restraint-Stressed Mice

**DOI:** 10.1155/2015/675149

**Published:** 2015-02-01

**Authors:** Ying Cai, Yi-Fang Li, Lu-Ping Tang, Bun Tsoi, Min Chen, Huan Chen, Xiao-Mei Chen, Rui-Rong Tan, Hiroshi Kurihara, Rong-Rong He

**Affiliations:** ^1^Anti-Stress and Health Research Center, Pharmacy College, Jinan University, Guangzhou 510632, China; ^2^Institute of Traditional Chinese Medicine & Natural Products, Pharmacy College, Jinan University, Guangzhou 510632, China

## Abstract

It is well known that vitamin C could protect against influenza infection, but little is known about the mechanisms. This study aimed to investigate the influence and possible mechanisms of vitamin C on pneumonia induced by influenza virus in stressed mice. Results showed that restraint stress significantly increased the mortality and the severity of pneumonia in mice caused by A/FM/1/47(H1N1) virus infection, which was attenuated by oral administration of vitamin C (125 and 250 mg/kg). Moreover, vitamin C administration significantly decreased expression of susceptibility genes, including mitochondrial antiviral signaling (MAVS) and interferon regulatory factor 3 (IRF3), and increased expression of NF-*κ*B. These work in conjunction to induce type I interferons (IFNs) and elicit innate antiviral response as key factors in RIG-I-mediated signal transduction pathway. The above effects of vitamin C were further found to relate with inhibition of excess CORT synthesis by regulating steroid hydroxylating enzymes in adrenal gland. In conclusion, the protective effects of vitamin C on influenza virus-caused pneumonia might be related to its inhibition of CORT synthesis, which reduces the susceptibility to influenza viral infection in restraint-stressed mice. These findings provide a new mechanism for the effects of vitamin C on influenza virus-induced pneumonia in restraint-stressed mice.

## 1. Introduction

Vitamin C is an essential antioxidant [1, 2] and an enzymatic cofactor for physiological reactions such as hormone production, collagen synthesis [3], and immune potentiation [4–6]. It has been studied for many years as a possible treatment for common colds [7, 8], which can increase the susceptibility to influenza. In 1961, Ritzel reported that children in a Swiss Alps ski school were treated with vitamin C (1 g/d), and they had a lower incidence and duration of influenza infection [9]. In the early 1970s, Pauling suggested that vitamin C (≥1 g/d) may substantially decrease the incidence and severity of common cold episodes [10]. Except for common cold, vitamin C was reported to relieve and prevent influenza symptoms when administered in megadoses before or after the appearance of symptoms in human study [11]. However, the mechanism of vitamin C effects on influenza is still unclear.

Influenza is an infection with a negative-stranded RNA virus that primarily infects the lung epithelium and leads to a strong local inflammatory response [12]. Influenza may evoke many symptoms and complications which eventually results in respiratory failure and death, such as chest pain, lymphadenopathy, and pneumonia. Influenza viruses can infect many people, especially susceptible crowd, such as newborns, the elderly, the sick, and individuals presenting fatigue or stress [13]. It is well known that psychological and physiological stress can activate the hypothalamic-pituitary-adrenal (HPA) axis and induce an excess of glucocorticoid (GC) [14–16]. Excessive GC caused by increased HPA activity can induce reactive oxygen species (ROS) production and result in inhibition of the susceptibility factors in the initiation of the innate antiviral immune response [17, 18]. Previous work from our laboratory has demonstrated that restraint stress increased susceptibility to viral or bacterial pathogens and influenced the severity of infectious disease [19, 20]. Moreover, influenza infection was reported to trigger a generalized stress response, leading to a sustained increase in serum GC level and a systemic suppression of host immune responses [21]. The key steps leading to the production of GC are undertaken by cytochrome P450 11h-hydroxylase (CYP11B1, also known as P45011h or P450c11), CYP17 (17a-hydroxylase/17,20-lyase, also known as P45017a or P450C17), and 21-hydroxylase cytochrome P450 (CYP21, also known as P450C21) [22]. It has been reported that vitamin C might play an important role in stress and response to GC [23]. Thus, the mechanism of vitamin C protective effects on influenza virus might relate with its antistress effects. In this study, the effects and possible mechanisms of vitamin C on pneumonia induced by influenza virus were investigated by restraint-stressed mouse model.

## 2. Materials and Methods

### 2.1. Materials and Chemicals

Vitamin C was purchased from Sigma-Aldrich (USA). Ribavirin was purchased from Biokin Pharmaceutical (Sichuan, China). Edaravone was purchased from Xiansheng Chemical (Jiangsu, China). CORT ((11*β*)-11,21-dihydroxypregn-4-ene-3,20-dione, CORT) was obtained from Sigma (St. Louis, USA). Methanol and ethyl acetate were purchased from Yuwang (Shandong, China). Phosphoric acid and sodium hydroxide were purchased from J. T. Baker Chemical Products Trading Co. Ltd. (Shanghai, China). The malondialdehyde (MDA) kit was purchased from Jiancheng Bioengineering Institute (Nanjing, China). Antibodies against MAVS, NF-*κ*B p65, and TNF-*α* were from Cell Signaling Technology Inc. (Boston, USA). Antibodies against iNOS and IRF3 were bought from Santa Cruz Biotechnology Inc. (CA, USA).

### 2.2. Animals

Male specific pathogen-free Kunming mice (13–15 g) were purchased from Guangdong Medical Laboratory Animal Center (Guangzhou, China). All mice were kept in a pathogen-free animal room under controlled temperature at 23 ± 1°C. A 12 h light-dark cycle was maintained, with lights on from 06:00 to 18:00. The mice were provided with a standard laboratory diet and water. The care and treatment of animals were conducted in accordance with the Guide for the Care and Use of Laboratory Animals in South China Agriculture University (SCAU-120017) as adopted and promulgated by the United States National Institutes of Health [19].

### 2.3. Virus

The influenza A/FM/1/47(H1N1) virus was provided by the College of Veterinary Medicine of South China Agricultural University (Guangzhou, China). The virus strain was propagated in specific pathogen-free fertilized eggs and adapted for lethality in mice after three passages in the animal. Virus-containing allantoic fluid was harvested and stored in aliquots at −80°C. The LD_50_ was determined in mice after serial dilution of the stock. Amounts equal to twice the LD_50_ value were used for viral challenge in all of the experiments. Infection was established by intranasal inoculation in mice anesthetized by ethyl ether. The influenza-related pathogenic operation was performed in the Animal Biosafety Level 3 Laboratory in South China Agriculture University.

### 2.4. Animal Experimental Design

The experimental mice were randomly divided into seven groups: normal group, virus group, model group, ribavirin group, edaravone group, vitamin C-low dosage group, and vitamin C-high dosage group. Model group mice were treated with restraint stress plus virus infection. Ribavirin (60 mg/kg) and vitamin C (125 or 250 mg/kg) were administered to mice by oral gavage and edaravone (3 mg/kg) was administered to mice by intraperitoneal injection for 7 consecutive days. The remaining groups received oral administration and intraperitoneal injection of water only. On the 1st day of administration, except for normal and virus groups, mice were physically restrained in a 50 mL polypropylene restraint tube with holes for 18 h. After recovering for three days, except for normal group, mice were anesthetized by inhalation of ether vapor and then an approximate 2x LD_50_ amount of virus (35 *μ*L) was instilled into the nares [19, 20]. The survival experiments were conducted on 10 mice from each group to observe daily changes in body weight, survival, and several typical symptoms of illness. These symptoms included ruffled fur, redness around the eyes, nose, or mouth, and hunched back; altered respiration; and unresponsiveness. The observations were continued for 21 days or until death. Another experiment was conducted in duplicate for mechanism study. On the 4th day after virus infection, these mice were weighed and anesthetized by ethyl ether. Under ethyl ether, blood was removed from the mice by cardiac puncture. Lungs and adrenal glands were harvested for analysis.

### 2.5. Cell Culture

A549 cells (human lung adenocarcinoma epithelial cell line) were provided by Guangzhou Medical University. A549 cells were maintained in Dulbecco's Modified Eagle Medium (DMEM) supplemented with 10% fetal bovine serum (FBS). Cells were maintained at 37°C in 5% CO_2_ to 80% confluence before being trypsinized and seeded into 6-well culture plates for experiments. The cells were treated with CORT (50 *μ*mol/L), CORT (50 *μ*mol/L) plus vitamin C (100 *μ*mol/L), respectively, for 24 h. Cells were collected and lysed for NF-*κ*B p65 protein expression determination.

### 2.6. Histopathologic Analysis

To monitor histological changes in the lungs of influenza virus-infected animals, all mice were weighed and sacrificed, and the lungs were removed and weighed. The lung index was calculated. Simultaneously, lung tissue was immediately fixed in 4% buffered formalin and embedded in paraffin wax. Lung transverse sections (thickness, 4 *μ*m) were sliced and mounted on microscopic slides. Histopathologic changes were examined by hematoxylin and eosin (H&E) staining. The changes of infiltration of inflammatory cells and thickened alveolar and bronchial walls were observed under a light microscope (Olympus, DP70).

### 2.7. Measurement of MDA Contents in Lungs

Lung tissue was homogenized in PBS. Lipid peroxidation in the lungs was determined by measuring thiobarbituric acid-reactive substances (TBARS) with a commercial MDA kit. In brief, 50 *µ*L of homogenate or an adequate volume of MDA working standard solution was introduced into 10 mL glass tubes containing 1 mL of distilled water. After addition of 1 mL of the solution containing 29 mmol/L TBA in acetic acid (pH of the reaction mixture, 2.4–2.6) and mixing, the samples were placed in a water bath and heated for 1 h at 95–100°C. After the samples cooled, 25 *µ*L of 5 mol/L HCl was added (pH 1.6-1.7), and the reaction mixture was extracted by agitation for 5 min with 3.5 mL of n-butanol. We separated the butanol phase by centrifugation at 1500 g for 10 min and measured the fluorescence of the butanol extract at wavelengths of 525 nm for excitation and 547 nm for emission.

### 2.8. Measurement of NO Level in Serum

Blood samples were collected and centrifuged at 500 g for 15 min to obtain serum. Serum NO levels were determined by the Griess test [19].

### 2.9. Determination of Mitochondrial Membrane Potentials

In this experiment, mitochondrial membrane potentials (Δ*ψ*m) of lung tissues were determined using a fluorescent probe, rhodamine-123 (Sigma, USA), a lipophilic cation that accumulates in the mitochondrial matrix in proportion to mitochondrial membrane potential. Lung tissues mitochondria suspensions were incubated with 10 *μ*M rhodamine-123 at 37°C for 30 min and then thoroughly washed three times with PBS. The fluorescence was determined using a flow cytometer.

### 2.10. Determination of CORT Levels in Plasma

Blood containing 100 U/mL heparin was transferred into 1.5 mL centrifuge tubes and the plasma was collected after centrifugation at 4500 g for 10 min. CORT was extracted from the plasma and quantified by HPLC using a modification of the method reported by Li et al. [24]. Plasma (0.5 mL) was mixed with 30 *µ*L of cortisone solution (0.125 mg/mL methanol-water 60 : 40 v/v) as an internal standard. Steroids were extracted by adding 2 mL of ethyl acetate and mixed thoroughly. The mixture was immediately centrifuged at 200 g for 5 min. The organic phase was washed twice with 1 mL of HPLC-grade water and centrifuged. The organic phase was then evaporated at room temperature under nitrogen. The residue was redissolved in 100 *µ*L of methanol-water (60 : 40, v/v) to measure the CORT level using HPLC with a UV detector at 254 nm (Hitachi, Japan). The column (5C18, 4.6 × 100 mm; particle size 5 *µ*m; Waters Corp., Milford, Massachusetts, USA) was equilibrated using HPLC-grade acetonitrile-water (38 : 72, v/v) at a flow rate of 1 mL/min.

### 2.11. Reverse Transcription Polymerase Chain Reaction (RT-PCR)

Total RNA in lysed lung tissue was extracted using Trizol Reagent according to the manufacturer's protocol (Invitrogen, Carlsbad, CA) and reversely transcribed to cDNA by applying mouse Moloney leukemia virus reverse transcriptase (Invitrogen, Carlsbad, CA). mRNA levels in lung tissue of MAVS, IRF3, NF-*κ*B, IL-6, IL-1*β*, CYP11B, CYP17A, and CYP21A and of the internal control 18S gene were measured by the Veriti PCR System (Applied Biosystems). The PCR products were fractionated on a 1% agarose gel and visualized by ethidium bromide staining. The band intensity of ethidium bromide fluorescence was measured using an image analysis system (Bio-Rad, Hercules, CA), then quantified with Quantity One analysis software (Bio-Rad, Hercules, CA), and expressed as the ratio to 18S. The sequences of the primers were as follows: 18S, 5′-AGGGGAGAGCGGGTAAGAGA-3′ and 5′-GGACAGGACTAGGCGGAACA-3′; MAVS, 5′-CAGATTGGTCCCAGTAA-3′ and 5′-GCAAGGTCCACAGAGC-3′; IRF3, 5′-AGAGGCTTGTGATGGT-3′ and 5′-GGCTGTTGGAGATGTG-3′; NF-*κ*B, 5′-TTTATCTCGCTTTCGG-3′ and 5′-GCTCCAGTCTGTCCCTC-3′; IL-1*β*, 5′-GCTGGAGAGTGTGGAT-3′ and 5′-CTTGTGAGGTGCTGATG-3′; IL-6, 5′-CCAACAGACCTGTCTATACCAC-3′ and 5′-GTGACTCCAGCTTATC-3′; CYP11B, 5′-CAGAACTAATGTGTATGT-3′ and 5′-TTGACCAGAGAAGATG-3′; CYP17A, 5′-CTGATACAAGCCAAGAT-3′ and 5′-CTGAAGCCTACATACTG-3′; CYP21A, 5′-CACTTCCTACAGCCTAA-3′ and 5′-CCTCCTCAATGGTTCT-3′.

### 2.12. Western Blotting

The protein was extracted from lung tissue and A549 cells by lysis buffer on ice for 5 min. After centrifugation at 12000 g for 10 min, the protein content of the supernatant was determined by a Pierce BAC Protein Assay Kit (Thermo Scientific, USA). Protein lysates were denatured with 5x loading buffer and separated on 10% or 15% SDS-polyacrylamide gel. After electrophoresis, protein bands were blotted onto a nitrocellulose membrane (Amersham Biosciences, Piscataway, USA). Proteins were detected using polyclonal antibodies to MAVS, NF-*κ*B p65, TNF-*α*, iNOS, and IRF3. These are visualized using anti-rabbit and anti-mouse IgG conjugated to horseradish peroxidase (HRP) and Pierce ECL Western Blotting Substrate (Thermo Fisher Scientific Inc., Santa Fe, USA) as the substrate of HRP.

### 2.13. Statistical Analysis

The data are presented as the mean ± standard error (SE). Statistical analysis of the data was performed using the SPSS 13.0 statistical package. One-way analysis of variance (ANOVA) was applied to analyze differences in data of biochemical parameters among the different groups. Differences were considered statistically significant at *P* < 0.05. Differences in morbidity and mortality between groups during the 21-day postinfection period were analyzed using the Life Test Survival Analysis program in SigmaStat, which analyzed mean and median time to sickness and death.

## 3. Results

### 3.1. Effects of Vitamin C on Morbidity and Mortality Caused by Influenza in Restraint-Stressed Mice

**Figure 1:**
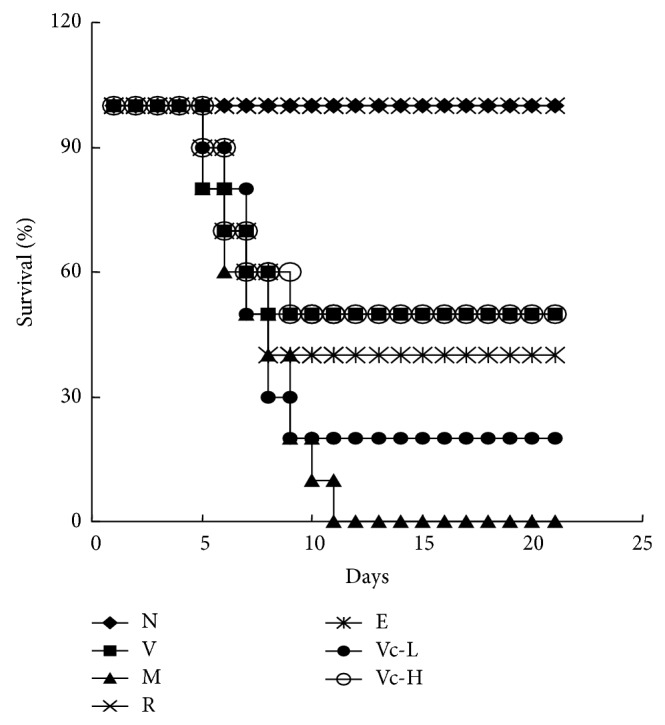
Effects of vitamin C on the survival rate of restraint-stressed mice after infection. Three days before H1N1 virus infection, Kunming mice were fixed in a restraint cage for 18 h. The time course of survival days was recorded until the 21st day after viral infection. Data were obtained from 10 animals in each group. N, normal; V, virus; M, model; R, ribavirin; E, edaravone; Vc-L, vitamin C-low dosage; Vc-H, vitamin C-high dosage.

After intranasal inoculation of A/FM/1/47(H1N1) influenza virus, the mice were monitored daily for survival, physical condition, and weight changes. Morbidity was defined as the onset day of sickness symptoms and presented as percentage of morbid mice to total number of mice. As a result, morbidity in the model group was 100%, while it was only 80% for the virus group. The mean time to sickness (MTS) was 6.2 ± 0.4 days for the model group and 9.2 ± 2.0 days for the virus group (*P* < 0.01). Vitamin C administration to mice at 125 and 250 mg/kg/day significantly alleviated symptom severity and offset morbidity (*P* < 0.05) and increased the MTS to 7.8 ± 1.5 and 9.4 ± 2.0 days, respectively. As shown in Figure 1, group differences in mortality were observed over the 21-day postinfection period. A significantly lower survival rate was observed in the model group as compared to the virus group (*P* < 0.01). None of the mice in the model group, loaded with restraint stress, survived and the mean day to death (MDD) was decreased from 13.9 ± 2.4 to 7.8 ± 0.8 days (*P* < 0.01). Ribavirin significantly increased the survival rate to 100%. Vitamin C also demonstrated inhibitory effects against virus-induced death, with 20% and 50% of mice surviving in the 125 and 250 mg/kg/day vitamin C treatment groups. The MDD of mice treated with 250 mg/kg/day vitamin C was prolonged to 13.8 ± 2.4 days (*P* < 0.05). These results indicated that vitamin C improved survival rates and prolonged survival time of virus-infected stressed mice in a dose-dependent manner.

### 3.2. Effects of Vitamin C on Lung Inflammation Caused by Influenza in Restraint-Stressed Mice

**Figure 2:**
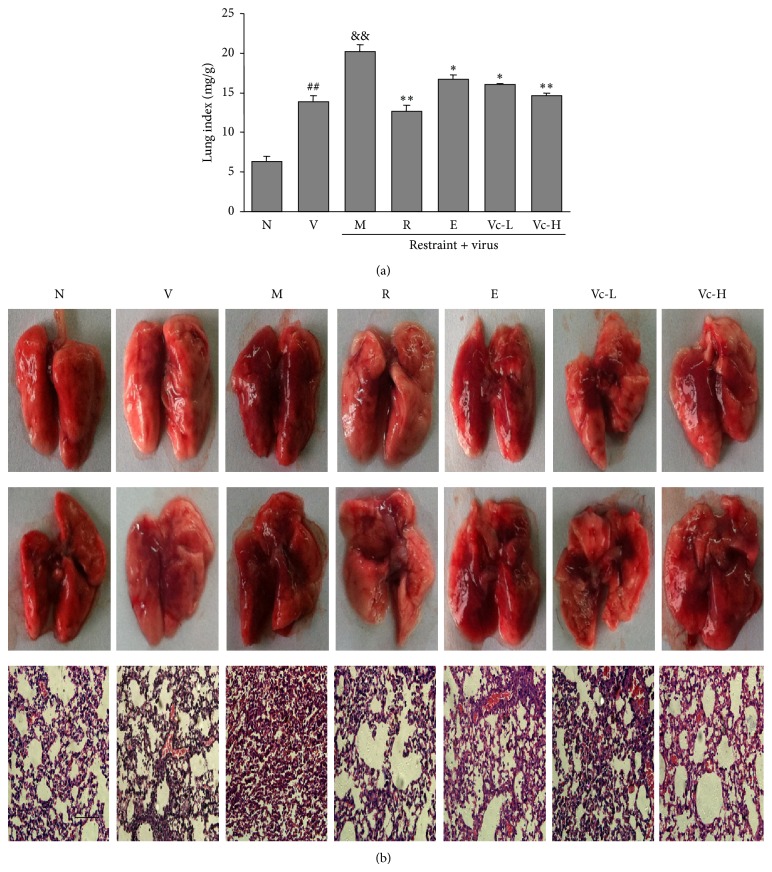
Effects of vitamin C on lung inflammation in restraint-stressed mice after infection. (a) Lung index was calculated according to the following formula: lung index = lung weight (mg)/body weight (g). The results represented the mean ± SE of values obtained from 10 mice in each group. (b) Histopathologic changes in lung tissue collected at the 4th day after infection. Representative histological sections of lung tissue from experimental mice were stained by H&E (magnification 400x, bar = 50 *μ*m). N, normal; V, virus; M, model; R, ribavirin; E, edaravone; Vc-L, vitamin C-low dosage; Vc-H, vitamin C-high dosage. Significant differences from the normal group at ^##^ *P* < 0.01, the virus group at ^&&^ *P* < 0.01, and model group at ^**^ *P* < 0.01, ^*^ *P* < 0.05.

The lung index was calculated as a parameter of pneumonia caused by the influenza virus. As shown in Figure 2(a), the baseline of the normal group is 6.3 ± 0.8 mg/g, while virus infection raised it to 13.8 ± 0.8 mg/g (*P* < 0.01) and further increased it to 20.2 ± 1.0 mg/g (*P* < 0.01) with restraint stress. In comparison with the model group, ribavirin significantly recovered the lung index to 12.6 ± 0.8 mg/g (*P* < 0.01). Moreover, the lung index was recovered to 16.0 ± 0.2 and 14.6 ± 0.4 mg/g (*P* < 0.01, *P* < 0.05) by low and high dosages of vitamin C, respectively. The effect of vitamin C on the histopathology changes of influenza virus-induced pneumonia in stressed mice was observed by H&E staining (Figure 2(b)). In lungs infected with influenza virus, infiltration of inflammatory cells and thickened alveolar and bronchial walls could be observed, especially in the model group. However, the lung morphology in the vitamin C and ribavirin groups showed less damage when compared with the model group.

**Figure 3:**
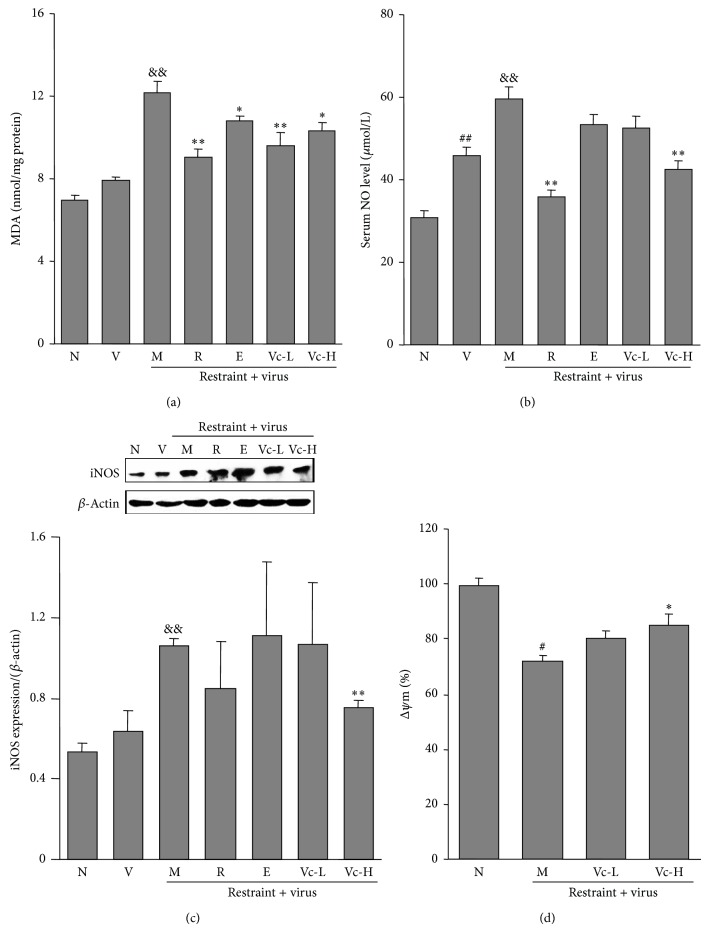
Effects of vitamin C on MDA content, serum NO level, iNOS protein expression, and mitochondrial membrane potential (Δ*ψ*m) of lung tissue in restraint-stressed mice after infection. (a) MAD contents in lung tissue; (b) NO level in serum; (c) iNOS protein expression intensity to *β*-actin of lung tissues; (d) mitochondrial membrane potential expressed as the ratio of fluorescent intensity to normal group. Kunming mice were administered with vitamin C (125 and 250 mg/kg) for 7 consecutive days starting 1 day before restraint stress. Mice were fixed in a restraint cage for 18 h and sacrificed 4 days later. The results represented the mean ± SE of values obtained from 10 mice in each group. N, normal; V, virus; M, model; R, ribavirin; E, edaravone; Vc-L, vitamin C-low dosage; Vc-H, vitamin C-high dosage. Significant differences from the normal group at ^##^ *P* < 0.01, ^#^ *P* < 0.05, the virus group at ^&&^ *P* < 0.01, and model group at ^**^ *P* < 0.01, ^*^ *P* < 0.05.

**Figure 4:**
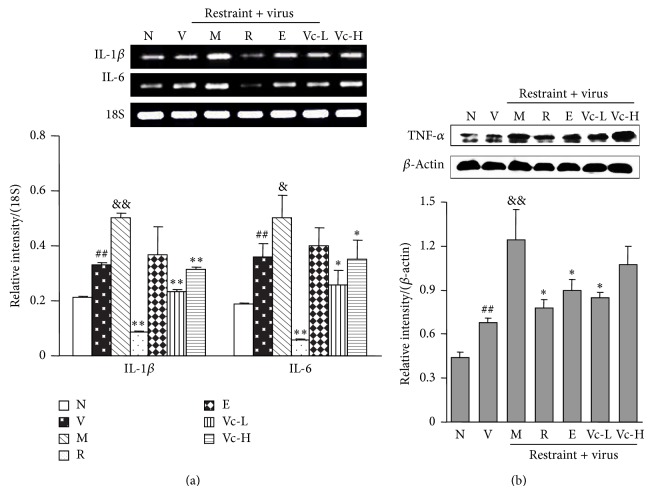
Effects of vitamin C on gene and protein expressions of proinflammatory cytokines in lung tissue of restraint-stressed mice after infection. (a) IL-1*β*, IL-6 mRNA expression in lung tissue from 10 mice in each group were determined by RT-PCR and normalized by 18S. (b) The protein levels of TNF-*α* in lung tissue were determined by western blotting and normalized by *β*-actin. N, normal; V, virus; M, model; R, ribavirin; E, edaravone; Vc-L, vitamin C-low dosage; Vc-H, vitamin C-high dosage. Significant differences from the normal group at ^##^ *P* < 0.01, from the virus group at ^&&^ *P* < 0.01, ^&^ *P* < 0.05, and from the model group at ^**^ *P* < 0.01, ^*^ *P* < 0.05.

To explore the effect of the inflammatory reaction in influenza-induced pneumonia, we measured the concentration of the nitrogen containing radical NO in serum (Figure 3(b)). The contents of lipid peroxidation and the protein level of iNOS in lung tissue were also determined (Figures 3(a) and 3(c)). As shown in Figure 3(a), the basal value of MDA was 6.9 ± 0.2 nmol/mg protein in the normal group mice. It was of markedly higher level in the model group mice (12.1 ± 0.6 nmol/mg protein, *P* < 0.01). Vitamin C groups (125 and 250 mg/kg) had less lipid peroxide in comparison with model group (9.6 ± 0.6 and 10.3 ± 0.5 nmol/mg, *P* < 0.01, *P* < 0.05, resp.). Also, as shown in Figures 3(b) and 3(c), serum NO level and the protein expression of iNOS were higher in the model group. Edaravone treatment did not significantly improve the above markers. However, oral administration of vitamin C could significantly reverse the elevations caused by restraint stress (*P* < 0.01). As shown in Figure 3(d), the level of mitochondrial membrane potential in the model group was markedly decreased compared to the normal group (*P* < 0.05). Administration of vitamin C significantly recovered the level of mitochondrial membrane potential (*P* < 0.05). At the same time, the levels of inflammatory markers in lung tissue such as IL-1*β*, IL-6, and TNF-*α* levels in lung tissues were also assayed. As shown in Figure 4, the gene expressions of IL-1*β* and IL-6 and the protein level of TNF-*α* in the model group were significantly increased as compared to the virus group (*P* < 0.01, *P* < 0.05). Administration of vitamin C could significantly decrease these gene and protein expressions (*P* < 0.05, *P* < 0.01). The above results indicated that vitamin C was more potent than edaravone for protection of the animal from influenza-caused pneumonia and inflammation caused under restraint stress.

### 3.3. Effect of Vitamin C on Plasma CORT Content in Influenza Infected Mice Loaded with Restraint Stress

**Figure 5:**
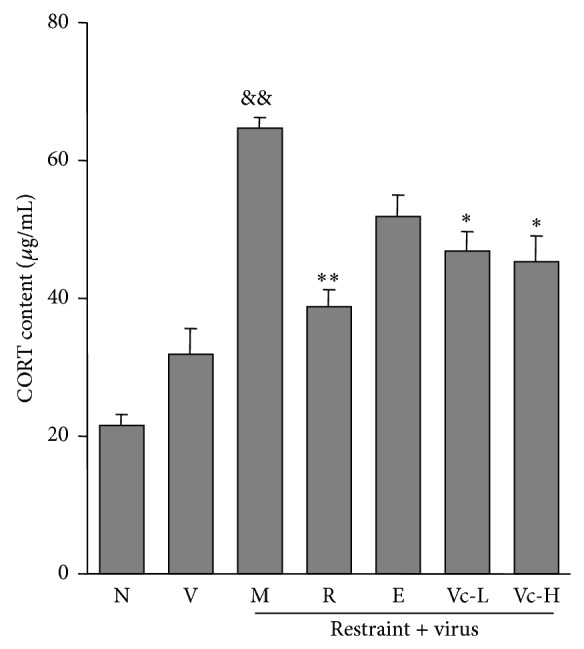
Effects of vitamin C on plasma CORT content in restraint-stressed mice after infection. N, normal; V, virus; M, model; R, ribavirin; E, edaravone; Vc-L, vitamin C-low dosage; Vc-H, vitamin C-high dosage. Significant differences from the virus group at ^&&^ *P* < 0.01 and from the model group at ^**^ *P* < 0.01, ^*^ *P* < 0.05.

As shown in Figure 5, the content of CORT in the plasma was increased in virus group and was further increased in the model group (*P* < 0.01). Administration of vitamin C significantly decreased the content of CORT in the plasma of influenza infected mice loaded with restraint stress (*P* < 0.05).

### 3.4. Effects of Vitamin C on Adrenal mRNA Expressions of CYP11B, CYP17A, and CYP21A in Restraint-Stressed Mice

**Figure 6:**
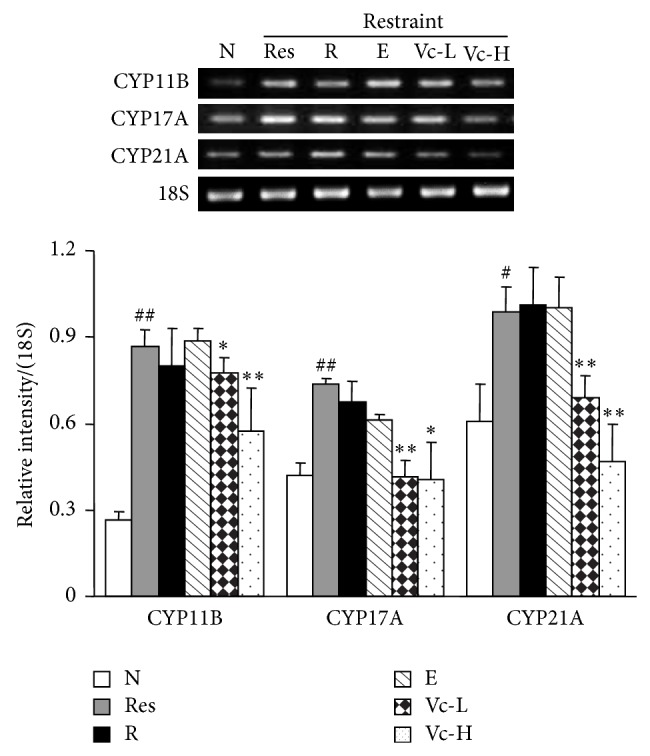
The effect of vitamin C on mRNA expression of CYP11B, CYP11A, and CYP21A in restraint-stressed mice after infection. The CYP11B, CYP11A, and CYP21A mRNA expression in adrenal tissues from mice were determined by RT-PCR and normalized by 18S. N, normal; Res, restraint; R, ribavirin; E, edaravone; Vc-L, vitamin C-low dosage; Vc-H, vitamin C-high dosage. Significant difference from the normal group at ^##^ *P* < 0.01, ^#^ *P* < 0.05 and from the restraint-stressed group at ^**^ *P* < 0.01, ^*^ *P* < 0.05.

The decrease in CORT content after vitamin C administration suggested a possible connection between CORT synthesis and the influence of vitamin C on restraint stress. In order to further understand this mechanism, the effects of vitamin C on the mRNA expression of the hydroxylases CYP11B, CYP17A, and CYP21A in the adrenal gland were determined in restraint-stressed mice. As shown in Figure 6, we found that CYP11B, CYP17A, and CYP21A mRNA expressions increased significantly in the model group (*P* < 0.01, *P* < 0.05). Vitamin C significantly decreased the mRNA expressions of CYP11B, CYP17A, and CYP21A in restraint-stressed mice (*P* < 0.01, *P* < 0.05), while ribavirin and edaravone had no obvious influence.

### 3.5. Effects of Vitamin C on mRNA and Protein Expressions of MAVS, IRF3, and NF-*κ*B p65 of Lung in Restraint-Stressed Mice

**Figure 7:**
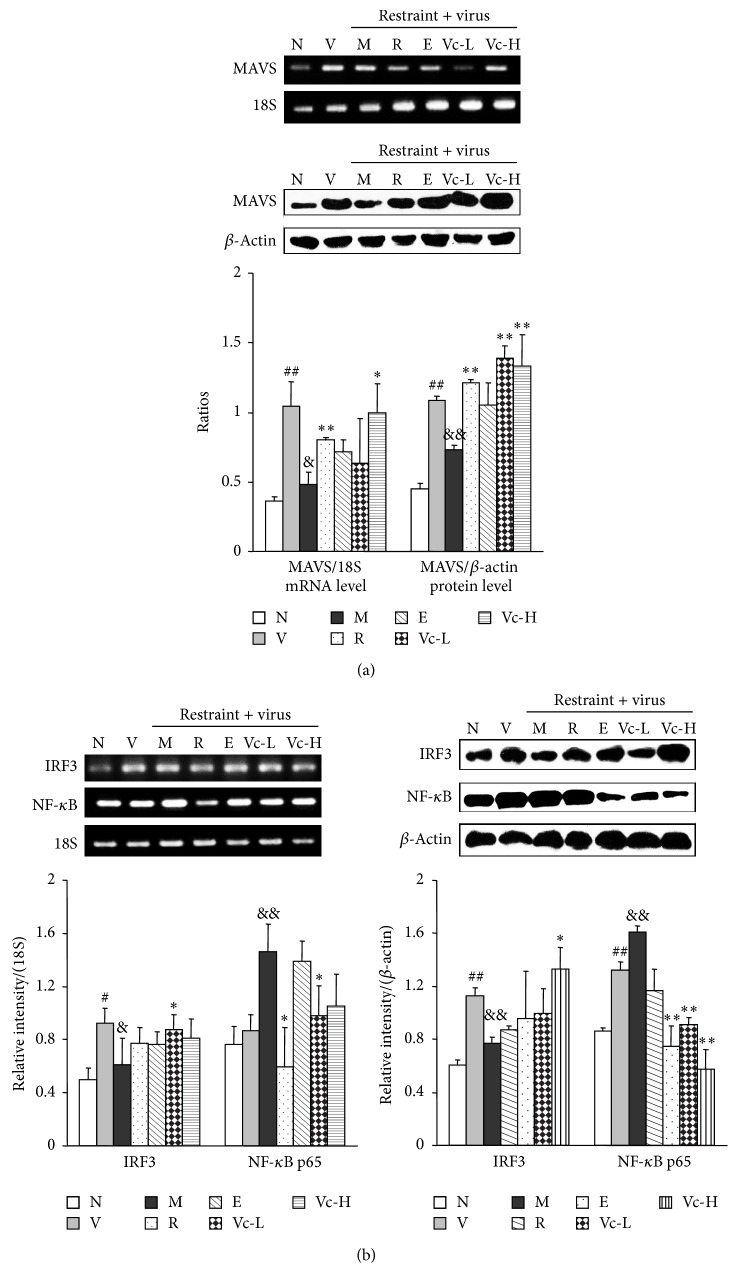
Effect of vitamin C on MAVS, IRF3, and NF-*κ*B p65 in lung tissues of restraint-stressed mice after infection. MAVS, IRF3, and NF-*κ*B p65 mRNA expression in lung tissues from 10 mice in each group were determined by RT-PCR and normalized by 18S. MAVS, IRF3, and NF-*κ*B p65 protein levels were determined by western blotting and normalized by *β*-actin. N, normal; V, virus; M, model; R, ribavirin; E, edaravone; Vc-L, vitamin C-low dosage; Vc-H, vitamin C-high dosage. Significant difference from the normal group at ^##^ *P* < 0.01, ^#^ *P* < 0.05, from the virus group at ^&&^ *P* < 0.01, ^&^ *P* < 0.05, and from the model group at ^**^ *P* < 0.01, ^*^ *P* < 0.05.

MAVS (mitochondrial antiviral signaling) plays a central role in virus-triggered activation of IRF3 and NF-*κ*B, which collaborate to induce IFNs and elicit an innate antiviral response. Therefore, the mRNA and protein expression levels of MAVS in the lung tissues were further examined. As shown in Figure 7(a), both gene and protein levels of MAVS in the model group were significantly decreased (*P* < 0.01), while vitamin C administration (250 mg/kg/day) significantly recovered MAVS gene and protein expressions (*P* < 0.01, *P* < 0.05). IRF3 and NF-*κ*B are crucial in host defense and could affect virus replication and clearance capability in the lungs. Therefore, the expression of IRF3 and NF-*κ*B in lung tissue was examined to further investigate the protective mechanism of vitamin C on influenza.  Figure 7(b) shows that the mRNA and protein expressions of IRF3 were significantly decreased in the model group compared to the virus group (*P* < 0.01, *P* < 0.05), and the expression of NF-*κ*B p65 was increased significantly (*P* < 0.01). Administration of vitamin C significantly increased the expression of IRF3 and decreased the expression of NF-*κ*B p65 in influenza infected mice loaded with restraint stress (*P* < 0.05, *P* < 0.01).

### 3.6. Effect of Vitamin C on Protein Expressions of NF-*κ*B p65 on CORT-Treated A549 Cells

**Figure 8:**
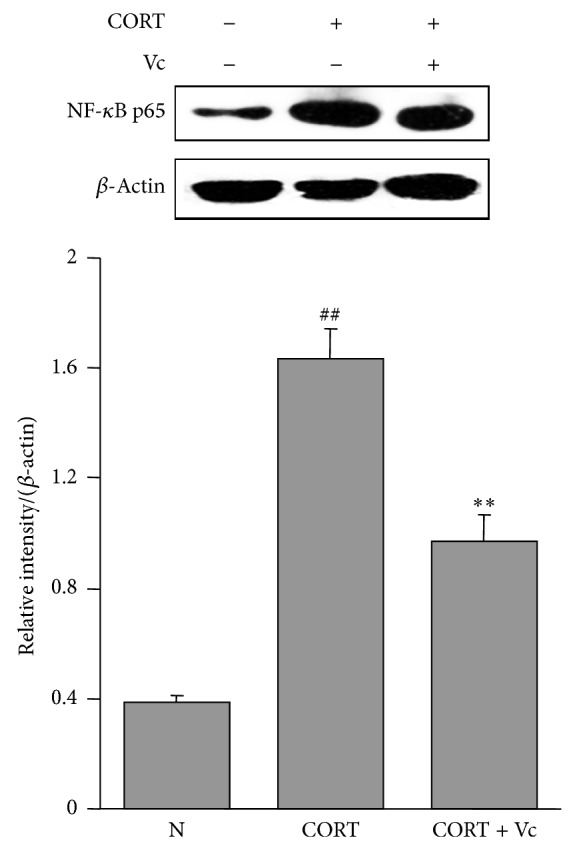
Effects of vitamin C on protein expression of NF-*κ*B p65 in CORT-treated A549 cells. NF-*κ*B p65 levels in A549 cells were determined by western blotting and normalized by *β*-actin. Significant difference from the normal group at ^##^ *P* < 0.01 and from the CORT group at ^**^ *P* < 0.01.

In order to explore the antivirus effect of vitamin C on susceptible factors, its influence on protein expression of NF-*κ*B p65 on CORT-treated A549 cells was determined. As shown in Figure 8, NF-*κ*B p65 protein expressions were significantly affected by the administration of CORT alone (*P* < 0.01). However, vitamin C treatment (Vc + CORT) significantly decreased the protein expression of NF-*κ*B p65 compared to the CORT group (*P* < 0.01).

## 4. Discussion

Although the effect of vitamin C on preventing influenza has been reported for many years [11], its underlying mechanism is still not clear. Vitamin C works in a very different way from traditional antiviral drugs in the human body. As a health supplement, vitamin C is thought to work in a synergic way that interacts with the virus and the body to keep the body in a fine balance [25]. Thus, we intend to explore the mechanism of vitamin C using a restraint-stressed mice model to simulate the susceptible population, which will consider both virus and host factors [26].

In the present study, the influence of vitamin C on influenza virus infection and the pneumonia was evaluated in restraint-stressed mice. The results show that intranasal administration of influenza virus could cause sickness in some of the animals. Although there were individual differences in experimental animals, the induction of sickness was found in all animals when the infected mice were loaded with restraint stress. These results correlate well with a previous study [27]. In comparison, the induction rate was only 80% for those without restraint stress. In a previous study, restraint stress had significantly altered the balance of CD4^+^/CD8^+^ T cells and reduced NK cell activity [19]. Obviously, restraint stress might affect susceptibility genes, which could elevate viral infection rate and induce sickness in mice. Therefore, a restraint-stressed mice model could be used to imitate the actual pathological condition of viral pneumonia. In our study, we found that oral administration of vitamin C (125 and 250 mg/kg) could elevate survival rates and prolong survival time in mice subjected to restraint stress before viral infection. Histopathology of lung sections also showed that fulminant viral pneumonia with substantial damage and severe inflammation in restraint stress-loaded infected mice. However, vitamin C treatment inhibited influenza virus-stimulated pulmonary morphological changes and decreased structural damage in blood vessel and alveoli. These results suggest that the effect of vitamin C in preventing influenza symptoms might be related to the amelioration of inflammation progression in the lungs. Previous work indicated that the levels of inflammatory cytokines in lung or serum appear to be closely correlated with the viral load [28]. Our results showed that inflammatory mediators (such as NO, iNOS, MDA, IL-6, IL-1*β*, and TNF-*α*) were increased significantly in the serum and lungs of restraint-stressed mice. This could be an indication sign of viral spread within the lungs. After oral administration of vitamin C, the levels of inflammatory cytokines were decreased at 4 days after infection. This result revealed that vitamin C prevented influenza virus infection and the subsequent pneumonia in restraint-stressed mice.

Viral infection triggers a series of signaling events that lead to induction of type I IFNs, including IFN-*β* and IFN-*α* family cytokines [29]. RIG-I (retinoic acid-inducible gene 1) and MDA5 (melanoma differentiation-associated protein 5) have been identified as a pattern-recognition receptor sensing intracellular dsRNA and were responsible for transmitting signals to downstream CARD-containing adaptor protein MAVS [30]. MAVS is the mitochondrial protein that activates NF-*κ*B and IRF3 transcription factors to induce IFNs and elicit an innate antiviral response [31]. Gene-deletion studies indicate that MAVS is required for the activation of IRF3 and NF-*κ*B, and MAVS-deficient mice exhibit severe deficiency in induction of type I IFNs and proinflammatory cytokines and are susceptible to RNA virus infection. The restored MAVS expression also facilitates the antiviral reaction in influenza infected stressed mice. In the present study, MAVS expression on the mitochondrial outer membrane was found significantly elevated by vitamin C administration on the 4th day after influenza infection. It is possible that MAVS allowed an early clearance of the virus and subsequently promoted an adaptive immune response. Interferon regulatory factor 3 (IRF3) is a transcription factor that is activated through MAVS. IRF3 is activated by phosphorylation of IRF3 which results in its nuclear accumulation, where it assembles with other transcription factors and contributes to the induction of the transcription of specific defense genes, including IFN-*β* [32]. IRF3 is essential for the immediate induction of transcription factors after virus infection. It is important in both the early and later phases of the antiviral immune response. NF-*κ*B is involved in the regulation of type I IFNs and cytokine-induced expression of inflammatory cytokines [33]. MAVS was activated and was phosphorylated by IKB kinase complex after viral infection and thus NF-*κ*B was activated by phosphorylated IKB [34]. Then NF-*κ*B is released into the nucleus and combines with IRF3 to initiate the transcription of IFN-*β* [35]. Our results showed that vitamin C administration can significantly increase the expression of IRF3 and decrease the expression of NF-*κ*B compared to the model group. This revealed that vitamin C prevented the viral pneumonia in restraint-stressed mice through lowering NF-*κ*B expression. Also, the IRF3 activation by vitamin C also decreased the susceptibility of the host to influenza virus, due to the initiation of downstream IFN-*β*.

GC is an important regulator at the end on the HPA axis, which mediates the homeostasis of the body and allows stress adaptation. CORT, one of the main GC found in rodents, is significantly released during a stress response [24]. In this study, we found that restraint stress promoted CORT secretion in mice, while the vitamin C treatment groups had a lower CORT level. GC biosynthesis is catalyzed by the action of a series of cytochrome P450 enzymes as well as reductases, such as CYP11B, CYP17, and CYP21 [36]. Our data demonstrate that vitamin C, but not ribavirin or edaravone, could suppress the mRNA expressions of CYP11B, CYP17A, and CYP21A in restraint-stressed mice. This suggests that the effect of vitamin C in influenza infected restraint-stressed mice is probably due to the inhibition of CORT synthesis, which is different from typical anti-influenza drugs and antioxidants. It can be inferred that the inhibition of CORT synthesis by vitamin C might increase the expression of the key factors of RIG-I-mediated signal transduction pathway to decrease the susceptibility to influenza viral infection.

It has been reported that an elevated amount of CORT could result in a significantly enhanced level of lipid peroxidation, suggesting an augmented production of ROS from the mitochondria [37]. Excessive ROS could affect the function of mitochondria, causing structural modification on the mitochondrial membrane [38]. During an antiviral reaction, MAVS must anchor on the mitochondrial outer membrane to mediate antiviral signaling pathway. Koshiba et al. have reported that MAVS does not combine with RIG-I-like receptors when the mitochondrial membrane potential was decreased, which hampered the antiviral signal transduction and thereby reduced the intensity of antiviral immune response [39]. In this study, mitochondrial membrane potential was lowered in mice of model group. Vitamin C administration was found effective in restoring mitochondria integrity. MAVS gene and protein expressions were also decreased in the model group and improved in vitamin C treatment groups as compared with the model group. These results suggested that the effect of vitamin C on CORT synthesis strengthened antioxidant ability and prevented mitochondrial damage. A previous study has suggested that CORT could interfere with the phosphorylation of IRF3 by inhibiting the interaction between a coactivator protein called GC receptor-interacting protein and IRF3 [18]. In our study, we found that vitamin C could prevent CORT-induced activation of NF-*κ*B p65 in A549 cells. Thus, it is inferred that vitamin C can inhibit GC caused changes of viral infection related factors in stressed mice.

Taken together, the protective effects of vitamin C on influenza virus-caused pneumonia might be related to its inhibition of CORT synthesis, which reduces the susceptibility to influenza viral infection in restraint-stressed mice. These results contribute to the understanding of the mechanism of vitamin C protection from influenza virus infection.
